# A Potassium Metal-Organic Framework based on Perylene-3,4,9,10-tetracarboxylate as Sensing Layer for Humidity Actuators

**DOI:** 10.1038/s41598-018-32810-7

**Published:** 2018-09-26

**Authors:** José Manuel Seco, Eider San Sebastián, Javier Cepeda, Blanca Biel, Alfonso Salinas-Castillo, Belén Fernández, Diego P. Morales, Marco Bobinger, Santiago Gómez-Ruiz, Florin C. Loghin, Almudena Rivadeneyra, Antonio Rodríguez-Diéguez

**Affiliations:** 1 Department of Applied Chemistry, Chemistry Faculty, The University of the Basque Country UPV/EHU, 20018 Donostia-San Sebastián, Spain; 2 Department of Electronics and Computer Technology, University of Granada, Granada, Spain; 3 Department of Analytic Chemistry, University of Granada, 18071 Granada, Spain; 4 Department of Inorganic Chemistry, University of Granada, 18071 Granada, Spain; 5 Pervasive Electronics Advanced Research Laboratory (PEARL), Department Electronics and Computer Technology, University of Granada, 18071 Granada, Spain; 6 Institute for Nanoelectronics, Technical University of Munich, Theresienstraße 90, N8, 1st floor DE-80333, Munich, Germany; 7 Department of Biology and Geology, Physics and Inorganic Chemistry, Universidad Rey Juan Carlos, C/ Tulipán s/n, 28933 Móstoles, Madrid Spain

## Abstract

We have synthesized a novel three-dimensional metal-organic-framework (MOF) based on the perylene-3,4,9,10-tetracarboxylate linker and potassium as metallic centre. We report the formation of this K-based MOF using conventional routes with water as solvent. This material displays intense green photoluminescence at room temperature, and displays an aggregation dependent quenching. Correlation of the optical properties with the crystalline packing was confirmed by DFT calculations. We also demonstrate its potential to build humidity actuators with a reversible and reproducible response, with a change of 5 orders of magnitudes in its impedance at about 40% relative humidity (RH). This 3D-MOF is based on an interesting perylene derivative octadentate ligand, a moiety with interesting fluorescent properties and known component in organic semiconductors. To the best of our knowledge, this is the first time to build such a printed and flexible actuator towards humidity with a reversible response, enabling precise humidity threshold monitoring.

## Introduction

**Figure 1:**
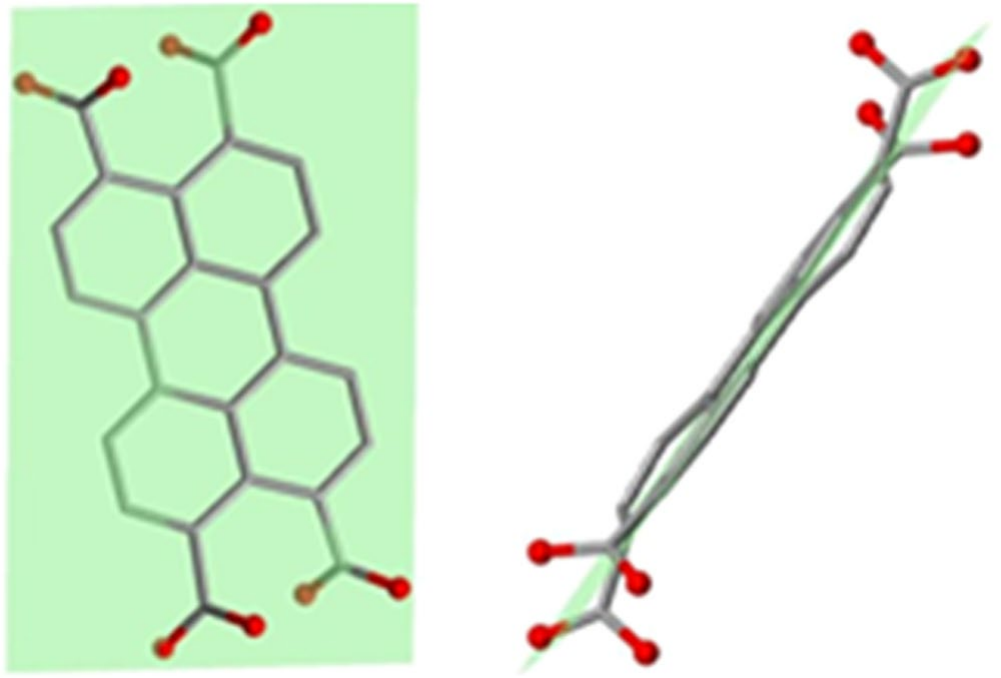
Different views of 3,4,9,10-perylenetetracarboxylic acid. Left: Normal view to the plane containing the perylenic unit. Right: View along the above plane.

Metal-organic frameworks (MOFs) are a relatively new class of materials that have attracted great interest due to their structural and topological diversity, as well as the properties^1–3^ that arise from their structural features. The combination of metal centres and organic ligands provides fantastic possibilities for the construction of materials with various structures and functionality^4–11^. The motivation of the present study arises not only from the limited number of s-block metal based MOFs reported to date, but from the variety of advantages^12^ that the use of these set of metal centres as building blocks of MOFs with distinct usability may imply, such as their low-cost relative to p/d-block metallic elements, their absence of toxicity, being essential in many biological processes, and, more importantly, their intrinsic ability to generate low density networks. In addition, it is known that s-block metals as dopants in MOFs may enhance their ability for gas storage due to the stronger binding capability of these metals to certain gases such as CO_2_ or H_2_^13^_._ Also, their complexes have shown a wealth of interesting properties, suitable for a range of applications from ferroelectrics to catalysts/nanozymes^14–19^. This motivations prompted us to synthesize new MOFs by using potassium as metal centre and to study some of the physical properties exhibited by these materials^20^. To construct novel potassium-based MOFs, 3,4,9,10-perylenetetracarboxylic acid (H_4_ptca) was chosen as a tetratopic linker, taking into account the interesting fluorescent and semiconducting properties that the perylene unit can exhibit^21^. Tetratopic carboxylate linkers appear to have great potential as building units in MOF construction, especially those with tetrahedral geometry^22^. Moreover, only one example of 2D-MOF constructed with potassium with this ligand is reported so far^23^. In our case, this linker shows a non-tetrahedral disposition due to the planarity of the perylenic unit (Fig. 1). Perylene displays blue fluorescence and is used as a blue-emitting dopant material in OLEDs and can be also used as an organic photoconductor^24^.

Owing to its extended aromaticity, (ptca)^4−^ is a good candidate for enhanced photoemission properties, which are tuneable by coordination to metal ions with different chemical environments. Herein, we report the synthesis, structure, luminescence measurements and DFT calculations of the 3D-MOF [K_8_(ptca)_3_(H_3_O)_4_]_n_ (**1**), demonstrating the potential of this tetracarboxylate linker to construct new MOFs with interesting physical properties.

Furthermore, we demonstrate the potential of this new MOF as sensing layer to build humidity actuators. In particular, we have deposited a layer of this compound on top of printed conductive electrodes on a flexible substrate to show its drastic change with moisture content from non-conductive device to a highly conductive one at a certain value of humidity. These results prove the potential of this kind of MOF to build flexible actuators with printed electronics with a very reproducible response. To the best of our knowledge, this is the first time to build such a printed and flexible actuator towards humidity.

## Results and Discussion

### Description of the structure

The conventional reaction of the appropriate amount of 3,4,9,10-perylenetetracarboxylic acid (1 mmol) with KOH (4 mmol) in water (40 ml) at 65 °C for 24 h produced prismatic orange crystals of **1**. The crystal structure was determined using single crystal X-ray crystallography. Compound **1** crystallizes in the trigonal space group *P*-3. The 3D-MOF structure of **1** is described by potassium atoms bridged by *(ptca)*^*4−*^ linkers and hydronium cations. In this MOF, K^+^ ions are connected by the eight oxygen atoms pertaining to the four carboxylate groups.

**Figure 2:**
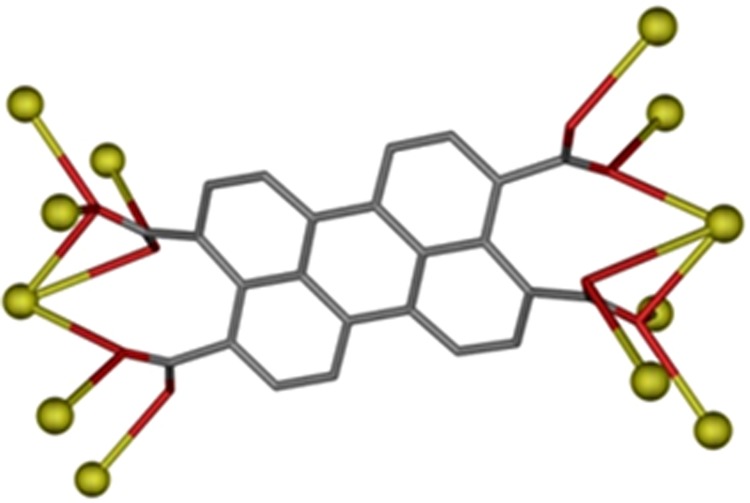
Coordination mode exhibited by 3,4,9,10-perylene-tetracarboxylate linker compound **1**.

**Figure 3:**
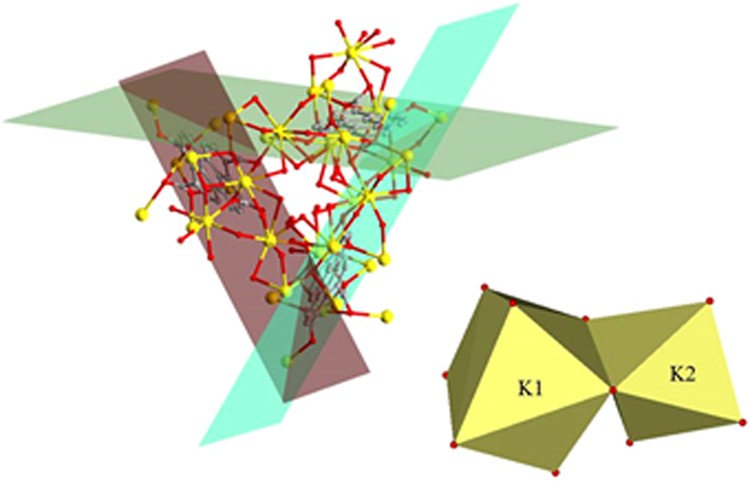
Left: Disposition of linkers in the structure. Right: Different coordination environments of K atoms. Hydrogen atoms have been omitted for clarity. O = red, C = grey, Dy = yellow.

The linker shows a coordination mode (Fig. 2) that bridges twelve potassium atoms. It should be noted that the presence of this coordination mode has not been reported previously for *(ptca)*^*4−*^ based coordination compounds. In the structure, the linkers are disposed forming dihedral angles of 64.08° among them, which generates hexagonal channels along c crystallographic axis (Fig. 3, left). These channels contain hydronium cations that could be interesting for photoconductance studies of this material.

**Figure 4:**
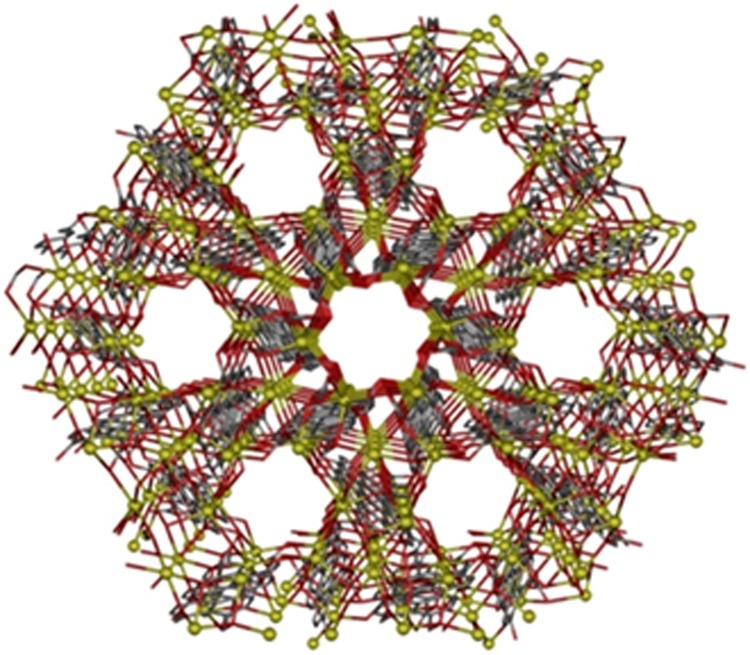
View down the *c* axis of the channels in compound **1**. Crystallization (H_3_O)^+^ molecules and hydrogen atoms have been omitted for clarity. Color code N = blue, O = red, C = grey, K = yellow.

The crystalline structure of this MOF is shown in Fig. 4. Interatomic distances and angles of ptca4-are within the range of values previously reported for perylene units^25^. The benzene ring is planar with the maximum deviation of 0.137 Å. Each plane of the carboxylate group is twisted out of the plane containing the perylene unit ca 48°. These twists and deviations might be due to the repulsion of the oxygen atoms of the adjacent carboxylate groups and to the formation of coordination bonds with potassium atoms. The C-O_carb_ distances in the present compound are in the range of 1.243(4)–1.261(5) Å and are not significantly different to those published^26^.

Two different potassium atoms are present in the crystalline structure. K1 has a K_1_O_8_ coordination environment whereas K2 has a distorted octahedral geometry (Fig. 3, right). Distortion of the K_1_O_8_ coordination polyhedron is induced mainly by the small O1-K1-O2W angle with a value of 57.7(19)°. The coordination environment of K1 consists of six oxygen atoms pertaining to carboxylate groups and two coordination water molecules. On the other hand, the octahedral environment from K2 is constructed of six oxygen atoms pertaining to carboxylate groups. The 3D-MOF (Fig. 4) presents channels along *c* axis and contains hydronium cations inside.

Gas adsorption analysis of N_2_ at 77 K on the activated sample of **1** revealed no appreciable porosity.

### Luminescence properties

**Figure 5:**
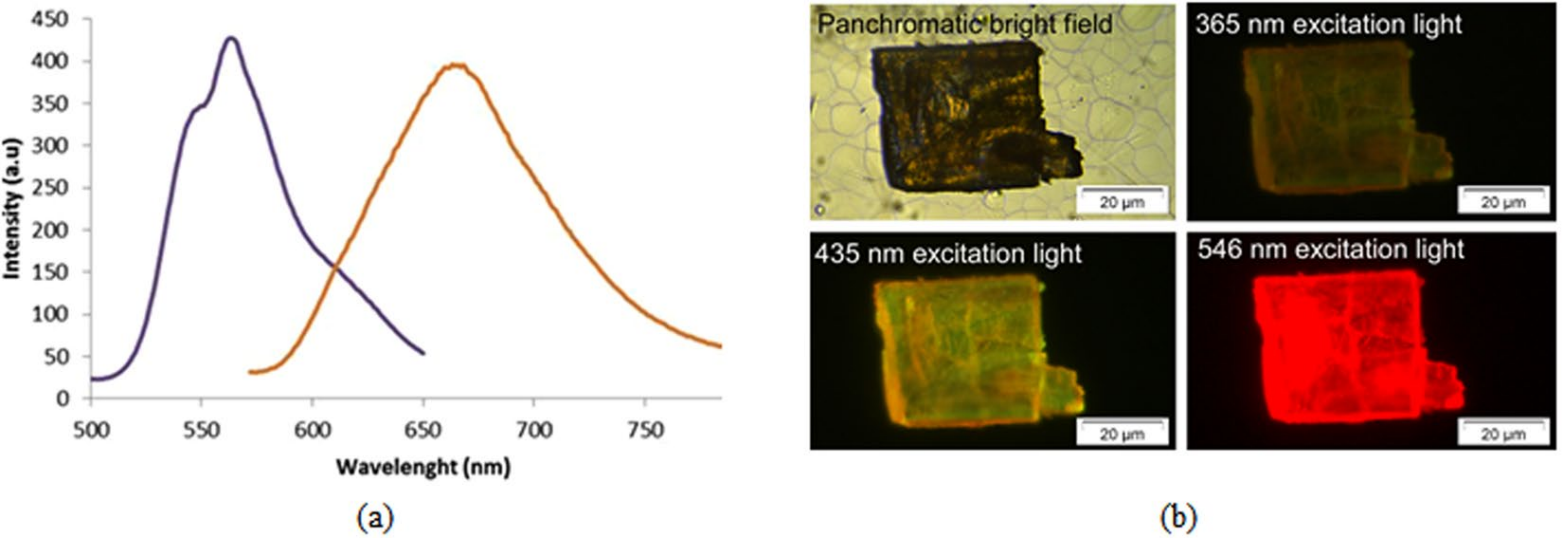
**(a)** The emission spectra of MOF (blue) and ligand (orange) after excitation at 410 and 585 nm, respectively, in solid state at room temperature. **(b)** Micro-PL images taken on a single crystal of compound **1**.

The conjugated π-systems of the perylene rings are of great interest in the field of fluorescent materials^27^. For this reason, we have studied the luminescence properties of **1** as well as the free ligand at room temperature. The H_4_ptca ligand displays a remarkably symmetric and broad emission band centred at λ_em_ = 665 nm upon excitation at λ_exc_ = 585 nm in the solid state, which is largely bathochromically shifted to give a narrower with two maxima at λ_em_ = 548 and 565 nm upon excitation at λ_exc_ = 410 nm when ptca coordinates to potassium ions in compound **1** (Fig. 5a). It is worth mentioning that a less intense shoulder is also distinguished in the high wavelength range of emission spectrum of **1**. Similar values have been reported for other material containing H_4_ptca linker^23^. Micro-PL images taken on a dark yellow coloured single crystal of **1** concur with its spectrum (Fig. 5b). Though negligible emission is observed when illuminated at 365 nm light (far below the excitation maximum at λ_exc_ = 410 nm), greenish and red emissions are inferred from the photographs taken with 460 and 535 nm excitation beams, both of which are representative for band maxima and high wavelength shoulder, respectively. To get deeper insights into the solid photoluminescence of **1**, the decay curve was monitored at the emission maxima. The analysis of the curve by tail fitting using a bi-exponential expression reveals the occurrence of short- and long-lived components with τ_1_ = 10.6(1) μs and τ_2_ = 214(2) μs, which may be attributed to the lamp pulse and the emission of **1**, respectively (see Figure S5 in the Supporting Information).

**Figure 6:**
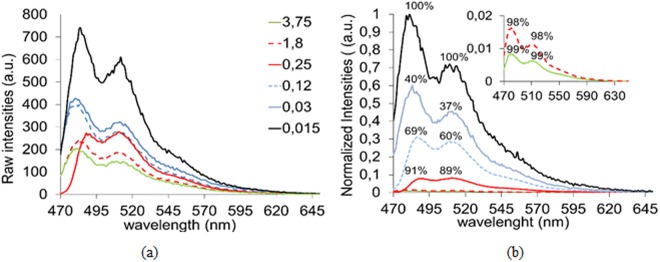
The emission spectra of water solutions of **1** at different concentrations (mM units) after excitation at 460 nm. (**a**) Raw data. (**b**) Data normalized according to the degree of dilution of each spectrum with respect to the spectrum at the highest concentration (3.75 mM). Above each maxima, the percentage of quenched signal is indicated.

Luminescence spectra were also recorded for water solutions of **1** at different concentrations (15 µM to 3.75 mM). Figure 6 (top) shows the obtained emission raw data; all spectra show two intense bands centred around 482 and 512 nm, respectively.

The former, may arise from π* → π electronic relaxations in the *(ptca)*^*4−*^ ligand, whereas the less energetic band at 512 nm could be related to electronic transitions involving the metal. The strongest emissions were observed for the most diluted sample (15 µM) which makes of this new material a highly efficient fluorophore both in solution and solid state. To gain some knowledge on the reasons promoting signal loss upon increasing the concentration of the samples, raw emission values where normalized considering the degree of dilution of each sample with respect to the most concentrated one (Fig. 6, bottom). Interestingly, at high concentrations (3,75 to 1,8 mM), both maxima are significantly quenched with respect to the most diluted spectrum, up to a 99% and 98%, respectively. In general, the lowest energy maxima are slightly less sensitive to concentration-dependent luminescence quenching than the highest energy ones. This dramatic quenching of luminescence signals may arise both from dynamic and/or static mechanisms^28^. Nevertheless, planar aromatic fluorophores like the *(ptca)*^*4−*^ linker are known to enhance static quenching, which arises from molecular aggregation that may occur in the ground state of highly concentrated samples.

**Figure 7:**
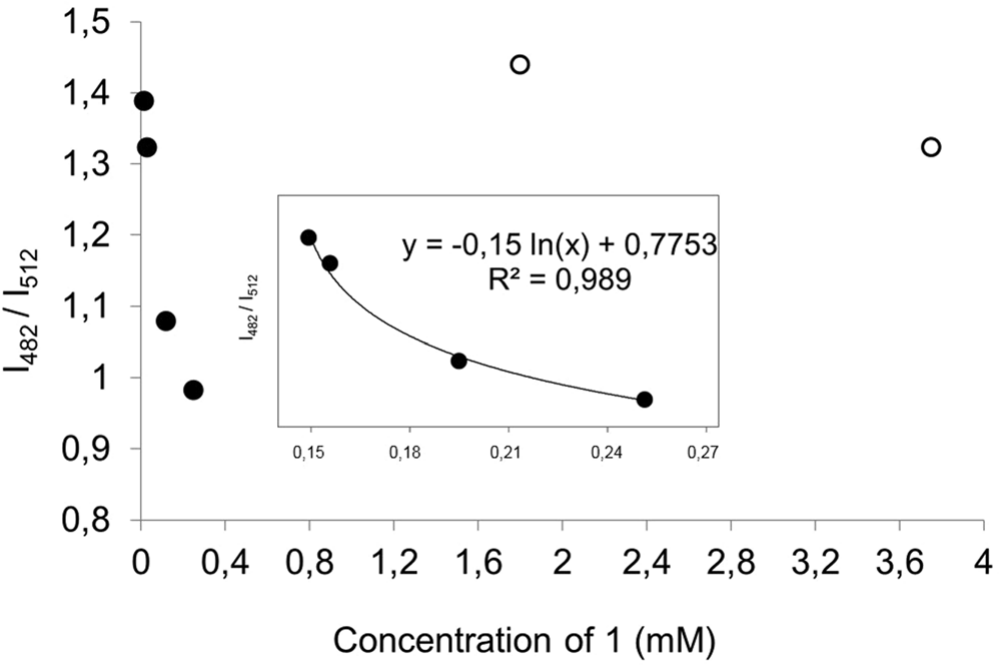
Ratio of the intensities of the maxima around 482 and 512 nm as a function of sample concentration. Logarithmic relationship between the decrease on the ratio of the intensities of each maximum and the increase of sample concentration from low (15 µM) to moderate (0.25 mM). Solid circles (low-to-moderate concentrations); Empty circles (high concentrations).

The effect of concentration on the ratio between the intensity values of each maximum was plotted in Fig. 7. A logarithmic relationship was found between the decrease on such ratio and the increase of sample concentration from low (15 µM) to moderate (0.25 mM). On the contrary, such relationship is lost at higher sample concentrations (empty circles in Fig. 7).

In this sense, we could consider two possibilities. If aggregation occurs via H-bonding or stacking interactions between *(ptca)*^*4−*^ linkers in adjacent MOF particles, as reported for other perylene derivatives^29^, it would be reasonable that the luminescence derived from π* → π electronic relaxations be quenched to a higher extent than the luminescence derived from charge-transfer processes involving the alkali metals and the carboxylate groups. On the other hand, this trend in quenching by aggregation is in agreement with previously reported studies on bare perylene^30^. The differences in the fluorescence profile of the MOF, the diluted, *(ptca)*^*4−*^, and the H_4_ptca in solid state indicate that the coordination to the K^+^ prevents the linkers from aggregation, thus leading to a shift in the fluorescence regarding to the protonated linker.

### Electronic structure simulations

Our preliminary Density Functional Theory (DFT)-based simulations confirm the stability of the compound and the preservation of the planarity of the benzene rings.

**Figure 8:**
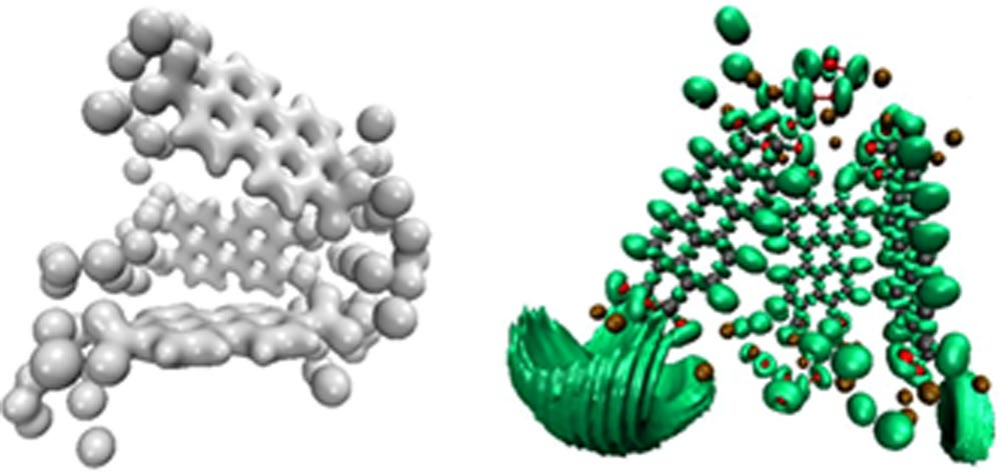
Left: Electronic charge density at an isosurface value of 0.7. Right: Electron Localization Function (ELF) at an isosurface value of 0.8.

The C-O distance ranges are between 0.125 and 0.129 Å, slightly above the experimental measurement as expected from Generalized Gradient Approximation calculations (GGA), which tend to overestimate length distances. The crystalline structure shown in Fig. 3 presents a direct GGA gap of around 1.68 eV. VMD^31^ was used to analyse and plot the electronic charge density and the Electron Localization Function (ELF) for both the isolated and the crystalline (not shown) structures. As expected the electronic charge tends to accumulate around the oxygen atoms (Fig. 8), with the highest values of the Electron Localization Function (ELF) obtained around the potassium atoms further away from the composite centre.

### K-Pery as sensing layer for humidity actuators

**Figure 9:**
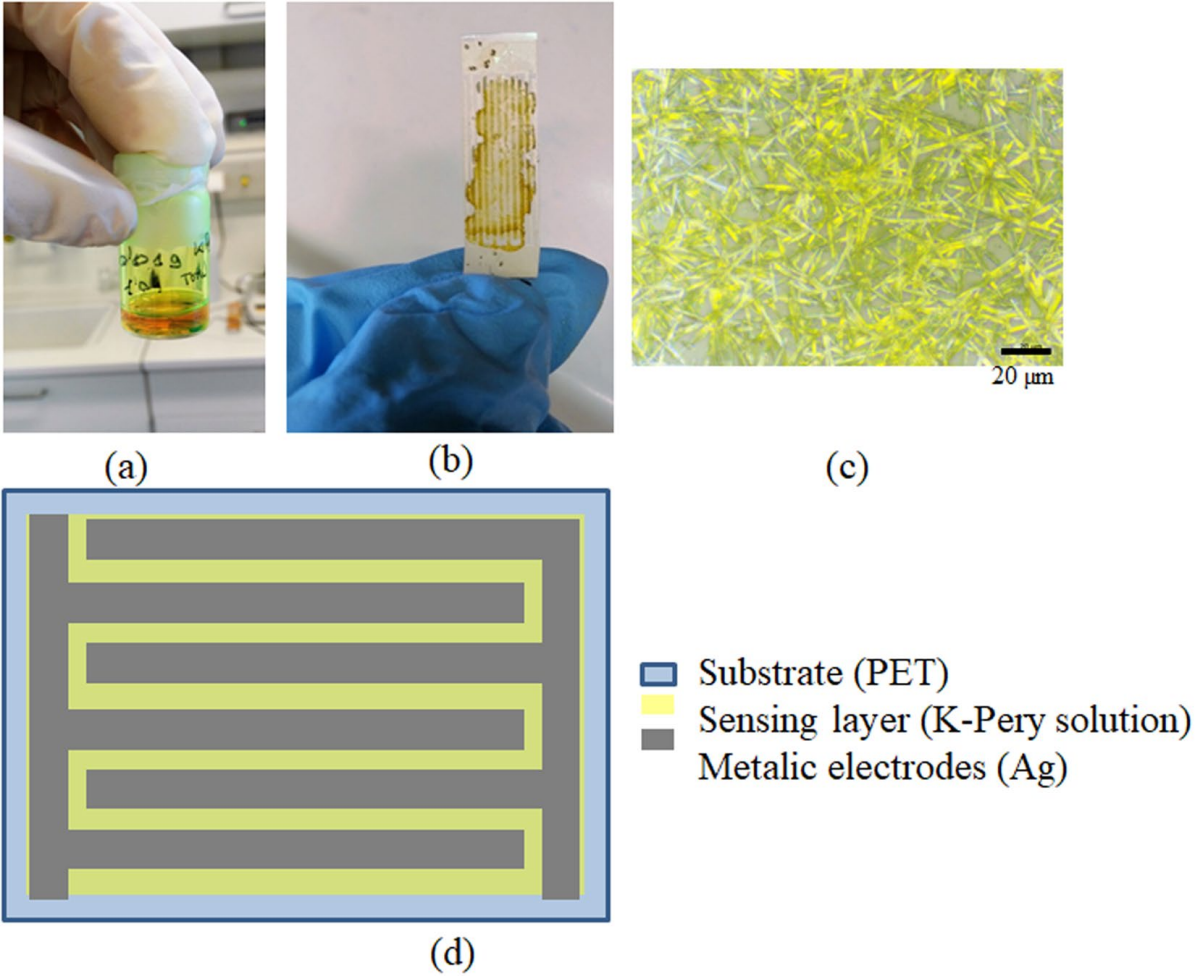
(**a**) K-Pery in water solution. (**b**) Solution drop-casted on top of silver IDEs. (**c**) Microscope image of the deposited K-Pery layer after drying. (**d**) schematic of the fabricated device.

The solution prepared to deposit the sensing layer is shown in Fig. 9a. The yellow colour exhibited by the solution is typically achieved by this kind of compounds. Figure 9b illustrates the manufactured device (2.2 cm × 0.9 cm). The electrodes (see Fig. 9d) are almost covered by the deposited layer of K-Pery. After drying, the compound precipitated forming the crystals shown in Fig. 9c.

**Figure 10:**
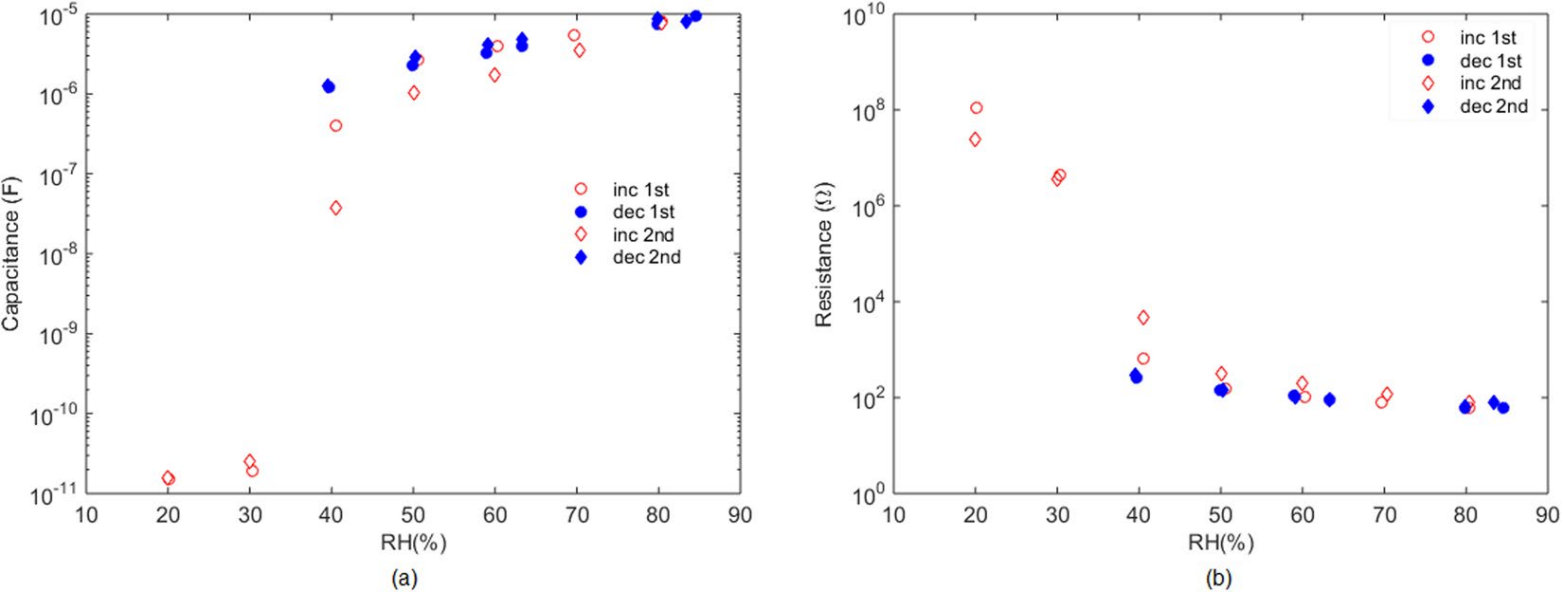
(**a**) Capacitance component of the device vs. RH at 100 Hz. (**b**) Resistive component of the device vs. RH at 100 Hz. Both graphs present two curves increasing and decreasing RH.

The characterization towards variation in moisture content of the printed device on a flexible substrate demonstrates the potential of this compound for relative humidity (RH) actuators. Figure 10 illustrates the behaviour of the fabricated device with RH. It can be observed that below 40%RH, its response is mainly capacitive (resistive component in the range of tens of MΩ) with a value of tens of pF. Above this RH value, the behaviour changes drastically. The sensor exhibits dominantly conductive behaviour: its resistive component decreases 5 orders of magnitude while the capacitive component goes to µF range.

This response could be exploited as a RH actuator, especially interesting for packaging to avoid spoiled products. Although this is a proof of concept, it shows the potential of this material for sensing and actuating purposes, particularly interesting its reversible behaviour.

**Figure 11:**
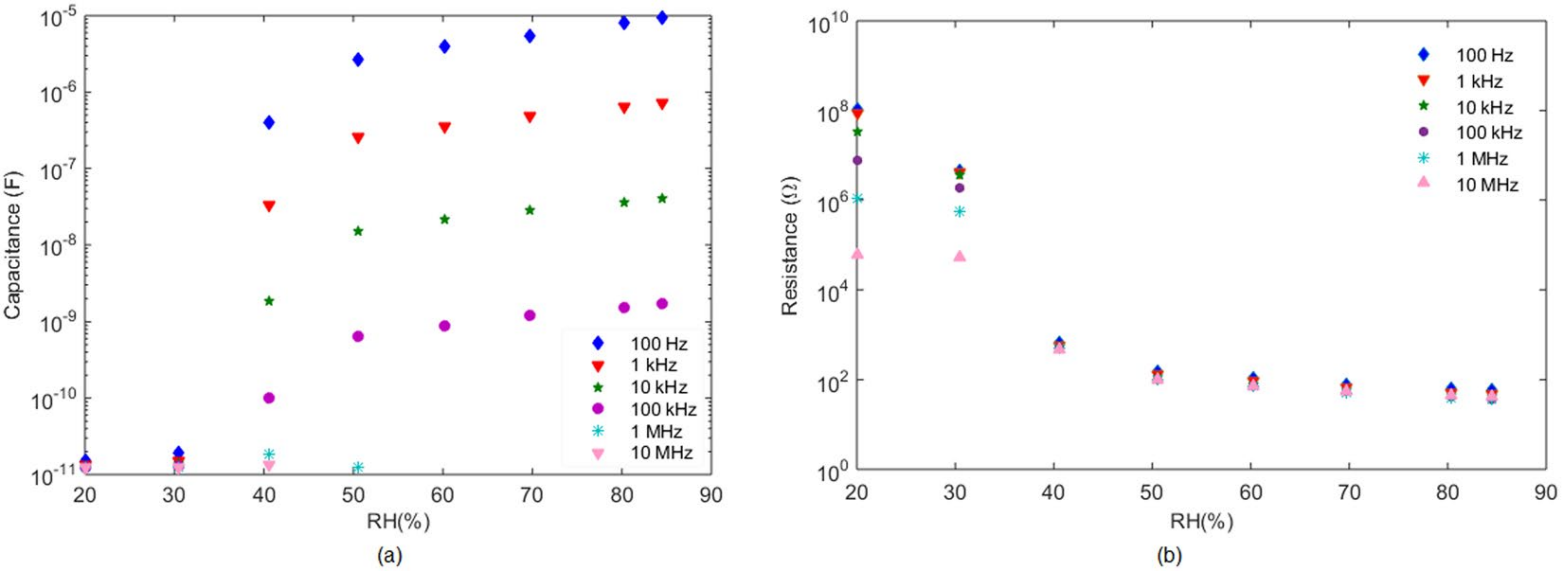
(**a**) Capacitance component of the device vs. RH(%) at different frequencies. (**b**) Resistive component of the device vs. RH% at different frequencies. Measurements performed at 25 °C.

Regarding the behaviour of the device in frequency regime (Fig. 11), the resistive component shows virtually no change in the range of frequencies studied whereas the capacitive part presents some differences. Although the shape of the response is the same in the range of RH analysed, its absolute value above 40%RH decreases drastically with the increase in frequency.

**Figure 12:**
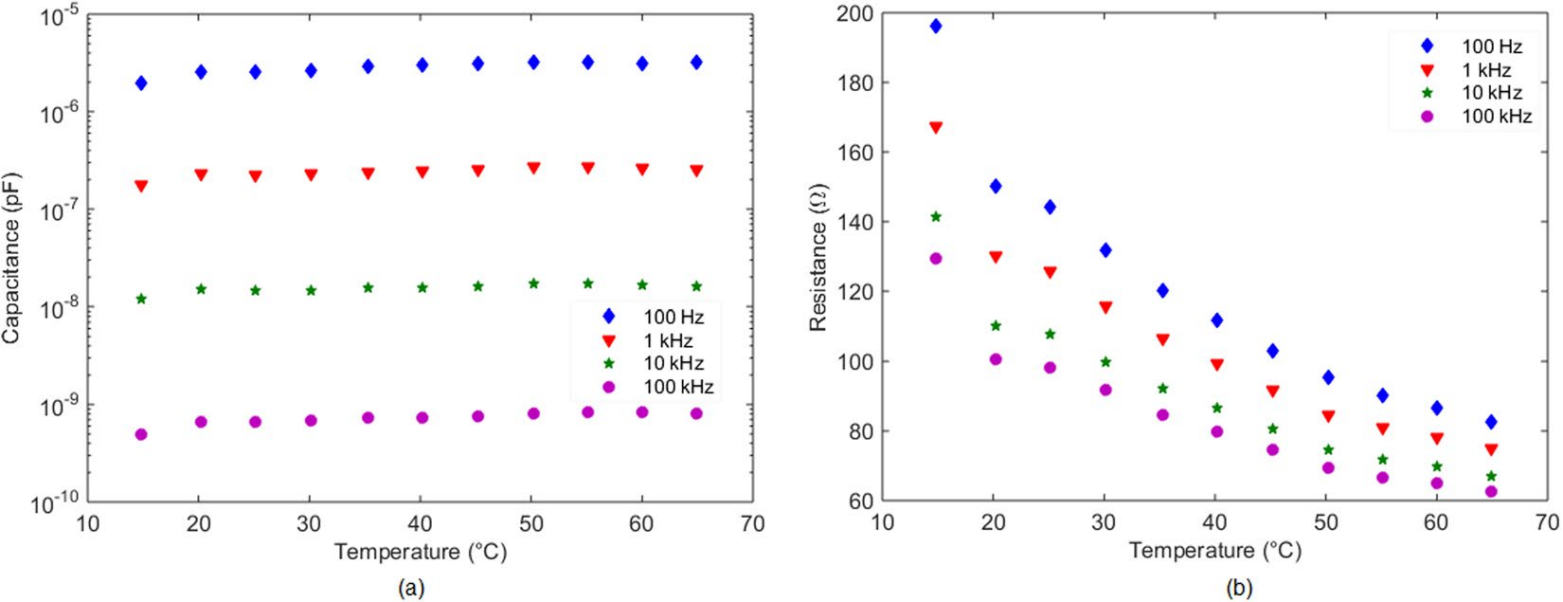
(**a**) Capacitance component of the device vs. Temperature at different frequencies. (**b**) Resistive component of the device vs. Temperature at different frequencies. Measurements performed at 60%RH.

Therefore, we can assume that the device can work below 1 MHz, showing the same binary states, both in its resistance and its capacitance parts (Fig. 12).

The variation in temperature at 20%RH shows virtually no difference whereas at 60%RH, where the sensitive layer has already become conductive, there is thermal drift (see Fig. 12): the higher the temperature is, the lower the resistance is obtained. This behaviour is virtually independent of the frequency. In particular, the resistance decreases from about 160 Ω at 25 °C (it remains constant at lower temperatures) to about 80 Ω at 60 °C (at higher temperatures) at 100 Hz. Virtually the same slope (2.28 Ω/°C) is found in the whole range of frequency analysed. Several printed humidity sensors have been already developed. Many of them are capacitive with a quite linear response in RH but there are also resistive sensors. The materials normally used are paper^32^, polyimide^33^ (both materials serve as sensing layer and substrate at the same time), cellulose acetate butyrate (CAB)^34^ or carbon nanotubes (CNTs)^35^. But they cannot be utilized as actuators because no abrupt change in their response occurs at any value of moisture content contrary to the here described device whose behavior varies from capacitive to resistive in less than 5%RH.

## Conclusion

A novel three-dimensional metal-organic-framework based on the perylene-3,4,9,10-tetracarboxylate linker and potassium as metallic centre has been synthesized and characterized. We also report the physical properties of this material and its preparation using conventional routes with water as solvent. This material displays intense photoluminescence properties in liquid and solid state at room temperature. Moreover, the potential of this compound to build humidity actuators on flexible substrates with printed techniques have been demonstrated. At low values of RH, the device shows a capacitive behavior, whereas after reaching a particular value of moisture (in this case, 40%RH), its impedance turns conductive with a very sharp response, essentially separating in the RH spectrum into two discerning states: a humidity switch. It is worth of mention, its reproducible behavior with very low hysteresis, features that make this MOFs a perfect candidate to build this kind of actuators. To the best of our knowledge, this is the first example of a 3D-MOF with this interesting octadentate ligand demonstrating the potential of this linker to construct MOFs with interesting and cutting-edge applications in nanotechnology.

## Experimental Section

### Materials and physical measurements

All reagents were obtained from commercial sources and used as received. Elemental (C, H, and N) analyses were performed on a Leco CHNS-932 microanalyzer. IR spectra of powder samples were recorded in the 400–4000 cm^−1^ region on a Nicolet 6700 FTIR spectrophotometer using KBr pellets.

1 $$Synthesis\,of\{{({H}_{3}O)}_{4}{[{K}_{8}{({\mu }_{12}-ptca)}_{3}{({H}_{2}O)}_{3}]}_{n}$$

Compound **1** was obtained by conventional routes through the following procedure: 0.008 g of 3,4,9,10-perylenetetracarboxylic acid (0.1 mmol) were added to 5 mL of H_2_O. The resulting solution was sonicated for 20 minutes, and then an aqueous solution (5 ml) containing KOH (0.1 mmol) was added. The reaction mixture was heated with IR light for 24 h. Orange prismatic crystals were obtained. Yields: 63% based on K^+^ ion. Elemental Analysis of C_72_H_36_K_8_O_28_ (**1**), calcd: C 52.04, H 2.18; found: C 52.31, H 2.09.

### Luminescence measurements

A Varian Cary-Eclipse Fluorescence Spectrofluorimeter was used to obtain the fluorescence spectra. The spectrofluorimeter was equipped with a xenon discharge lamp (peak power equivalent to 75 kW), Czerny-Turner monochromators, R-928 photomultiplier tube which is red sensitive (even 900 nm) with manual or automatic voltage controlled using the Cary Eclipse software for Windows 95/98/NT system. The photomultiplier detector voltage was 700 V and the instrument excitation and emission slits were set at 5 and 5 nm, respectively. A closed cycle helium cryostat enclosed in an Edinburgh Instruments FLS920 spectrometer was employed for lifetime measurements.

### Adsorption Analysis

N_2_ adsorption isotherms were undertaken at 77 K using a Micromeritics ASAP 2020 instrument. Samples were activated at 393 K for 8 hours previous to the adsorption measurement.

### Crystallographic refinement and structure solution

Prismatic crystals for **1** were mounted on a glass fibre and used for data collection on a Bruker D8 Venture with Photon detector equipped with graphite monochromated MoKα radiation (λ = 0.71073 Å). The data reduction was performed with the APEX2^36^ software and corrected for absorption using SADABS^37^. Crystal structures were solved by direct methods using the SIR97 program^38^ and refined by full-matrix least-squares on *F*^2^ including all reflections using anisotropic displacement parameters by means of the WINGX crystallographic package^39,40^. Generally, anisotropic temperature factors were assigned to all atoms except for hydrogen atoms, which are riding their parent atoms with an isotropic temperature factor arbitrarily chosen as 1.2 times that of the respective parent. It should be noted that all crystals diffract with low quality of the data. We measured eight different crystals of this material and the structure was solved from the best data we were able to collect. Moreover, to solve the problems present in this structure we collected in triclinic mode the data pertaining to this compound. Final R(F), wR(F2) and goodness of fit agreement factors, details on the data collection and analysis can be found in Table 1. CCDC numbers is 1509483. These data can be obtained free of charge from The Cambridge Crystallographic Data Centre via www.ccdc.cam.ac.uk/data_request/cif.

**Table 1:** Crystallographic data and structure refinement details of compound 1.

Compound	1
Chem. form.	C_72_H_36_K_8_O_28_
Form. Weight	1649.71
Cryst. System	Trigonal
Space group	*P*-3
*a* (Å)	12.1841(6)
*b* (Å)	12.1841(6)
*c* (Å)	14.0000(7)
V (Å^3^)	1799.9(2)
Z	1
*ρ* (g cm^−3^)	1.533
*μ* (mm^−1^)	0.565
Unique reflections	40355
R_int_	0.046
GOF^a^	1.040
R_1_^b^/wR2^c^[I > 2σ(I)]	0.0765/0.1699
R_1_^b^/wR2^c^ (all data)	0.0872/0.1766

^a^S = [∑w(F_0_^2^ − F_c_^2^)^2^/(N_obs_ − N_param_)]^1/2^.

^b^R_1_ = ∑||F_0_| − |F_c_||/∑|F_0_|.

^c^wR_2_ = [∑w(F_0_^2^ − F_c_^2^)^2^/∑wF_0_^2^]^1/2^.

### Theoretical section

The electronic structure of the compound was analysed by means of Density Functional Theory. Simulations of the structure as provided by X-Ray measurements were performed using the plane waves VASP (Vienna Ab initio Simulation Package)^41–43^, within the pseudopotential approximation and the Projector Augmented Wave (PAW) approach. Van der Waals interactions were included through the Klimes parametrization^44,45^, and the energy cut-off of the plane waves was set to 450 eV, the highest default value as given by the pseudo-potentials provided by VASP. We employed a vacuum layer for the supercell calculations of 18 Å to avoid undesired interaction between neighbouring supercells.

### Scanning electron microscopy

Scanning electron microscopy (SEM) was performed. SEM-images were recorded with a field-emission scanning electron microscope (NVision40 from Carl Zeiss) at an extraction and acceleration voltage of 1 kV. To optimize the image quality, the working distance was adjusted in the range 5–6 mm.

### Device fabrication

First the K-Pery powder was dissolved in water (1:10) and sonicated for 15 min. Then, it was drop casted on top of a polyethylene terephthalate (PET) substrate. Before drop casting, we defined silver interdigitated electrodes via screen printing (Siebdruck-Versand, Germany) using silver conductive paste (Sigma Aldrich, USA). During the deposition of K-Pery solution, the substrate was set to 80 °C to facilitate the evaporation of the water. The total area of the sensor is 2.2 × 0.9 cm with an interspace and finger width of 500 µm and 4 fingers per electrode.

### Device characterization

The sensor was characterized using an impedance analyzer (E4990A, Keysight Technologies) with the probe 4294A1 (Keysight Technologies) in the frequency range from 100 Hz to 10 MHz. The excitation signal was V_AC_ = 500 mV and V_DC_ = 0 V. The sensor was placed in a climatic chamber (VLC4006, Vöscht). Humidity tests were performed at 40 °C, increasing and decreasing from 20%RH to 90%RH in 10%RH steps every 1 h to ensure a stable value in the chamber. Temperature measurements were done from 25 °C to 70 °C at 20%RH and from 10 °C to 70 °C at 55%RH. The difference in the temperature range comes from constrains in the climatic chamber. In all cases the temperature step was 5 °C every 30 min, ensuring a constant temperature in the chamber.

## Electronic supplementary material

Supporting Information

Compound 1
