# Supplementary material for: A Potassium Metal-Organic Framework based on Perylene-3,4,9,10-tetracarboxylate as Sensing Layer for Humidity Actuators
Source: Sci Rep. 2018 Sep 26;8:14414. doi: 10.1038/s41598-018-32810-7 (PMC6158245; doi:10.1038/s41598-018-32810-7)
Supplement: Supplementary file 1 — Supporting Information [file 41598_2018_32810_MOESM1_ESM.docx]

A Potassium Metal-Organic Framework based on Perylene-3,4,9,10-tetracarboxylateas Sensing Layer for Humidity Actuators

José Manuel Seco, Eider San Sebastián, Javier Cepeda, Blanca Biel, Alfonso Salinas-Castillo, Belén Fernández, Diego P. Morales, Marco Bobinger, Santiago Gómez-Ruiz, Florin C. Loghin, Almudena Rivadeneyra* and Antonio Rodríguez-Diéguez *

**Supporting Information**

*Crystallographic Table*

**Table S1**.Bond distances (Å) and angles (°) for compound **1**

| Bond distances | Bond angles | Bond angles |
| --- | --- | --- |
| K1 O3 2.706(3)  K1 O2 2.732(4)  K1 O4 2.738(2)  K1 O2 2.796(3)  K1 O1 2.906(2)  K1 C1 3.185(3)  K1 O1 3.284(3)  K1 O1W 3.3(4)  K1 C12 3.463(3)  K1 C2 3.525(3)  K1 C1 3.526(4)  K1 K2 3.8198(10)  K2 O4 2.660(2)  K2 O4 2.660(2)  K2 O4 2.660(2)  K2 O2 2.780(3)  K2 O2 2.780(3)  K2 O2 2.780(3)  K2 K1 3.8198(10)  K2 K1 3.8198(10)  K2 K1 3.8198(10)  K2 K1 4.3919(10)  K2 K1 4.3919(10)  K2 K1 4.3919(10) | O3 K1 O2 124.23(9)  O3 K1 O4 154.11(10)  O2 K1 O4 80.89(8)  O3 K1 O2 102.97(10)  O2 K1 O2 83.63(10)  O4 K1 O2 71.06(8)  O3 K1 O1 81.78(9)  O2 K1 O1 102.22(8)  O4 K1 O1 100.19(8)  O2 K1 O1 168.80(9)  O3 K1 C1 111.09(9)  O2 K1 C1 96.20(9)  O4 K1 C1 54.39(8)  O2 K1 C1 23.08(9)  O1 K1 C1 145.72(9)  O3 K1 O1 100.11(8)  O2 K1 O1 117.71(7)  O4 K1 O1 58.10(7)  O2 K1 O1 41.94(8)  O1 K1 O1 127.62(9)  C1 K1 O1 22.23(8)  O3 K1 O1W 69(4)  O2 K1 O1W 148(5)  O4 K1 O1W 93(3)  O2 K1 O1W 125(4)  O1 K1 O1W 47(4)  C1 K1 O1W 106(3)  O1 K1 O1W 84(3)  O3 K1 K2 158.50(7)  O2 K1 K2 46.66(6)  O4 K1 K2 44.14(5)  O2 K1 K2 95.50(7)  O1 K1 K2 82.09(6)  C1 K1 K2 90.19(6)  O1 K1 K2 100.98(5)  O1W K1 K2 109(5)  O4 K2 O4 103.02(6)  O4 K2 O2 84.95(8)  O4 K2 O2 81.38(9)  O4 K2 O2 169.56(8)  O4 K2 O2 81.38(9)  O4 K2 O2 169.56(8)  O4 K2 O2 84.95(8)  O2 K2 O2 89.66(8)  O4 K2 O2 169.56(8)  O4 K2 O2 84.95(8) | O4 K2 O2 81.38(9)  O2 K2 O2 89.66(8)  O4 K2 K1 45.79(5)  O4 K2 K1 132.19(7)  O4 K2 K1 117.34(6)  O2 K2 K1 63.60(7)  O2 K2 K1 45.61(7)  O2 K2 K1 123.78(6)  O4 K2 K1 117.34(6)  O4 K2 K1 45.79(5)  O4 K2 K1 132.19(7)  O2 K2 K1 45.61(7)  O2 K2 K1 123.78(6)  O2 K2 K1 63.60(7)  K1 K2 K1 109.21(2)  O4 K2 K1 132.19(7)  O4 K2 K1 117.34(6)  O4 K2 K1 45.79(5)  O2 K2 K1 123.78(6)  O2 K2 K1 63.60(7)  O2 K2 K1 45.61(7)  K1 K2 K1 109.21(2)  K1 K2 K1 109.21(2)  O4 K2 K1 57.75(6)  O4 K2 K1 66.72(6)  O4 K2 K1 152.28(6)  O2 K2 K1 38.16(5)  O2 K2 K1 109.04(7)  O2 K2 K1 121.26(7)  K1 K2 K1 65.64(3)  K1 K2 K1 59.74(3)  K1 K2 K1 161.93(3)  O4 K2 K1 66.72(6)  O4 K2 K1 152.28(6  O4 K2 K1 57.75(6)  O2 K2 K1 121.26(8)  O2 K2 K1 38.16(5)  O2 K2 K1 109.04(7)  K1 K2 K1 161.93(3)  K1 K2 K1 65.64(3)  K1 K2 K1 119.886(2  O4 K2 K1 66.72(6)  O2 K2 K1 109.04(7)  O2 K2 K1 121.26(7)  O2 K2 K1 38.16(5)  K1 K2 K1 161.93(3)  K1 K2 K1 65.64(3) |

***PXRD characteristic***


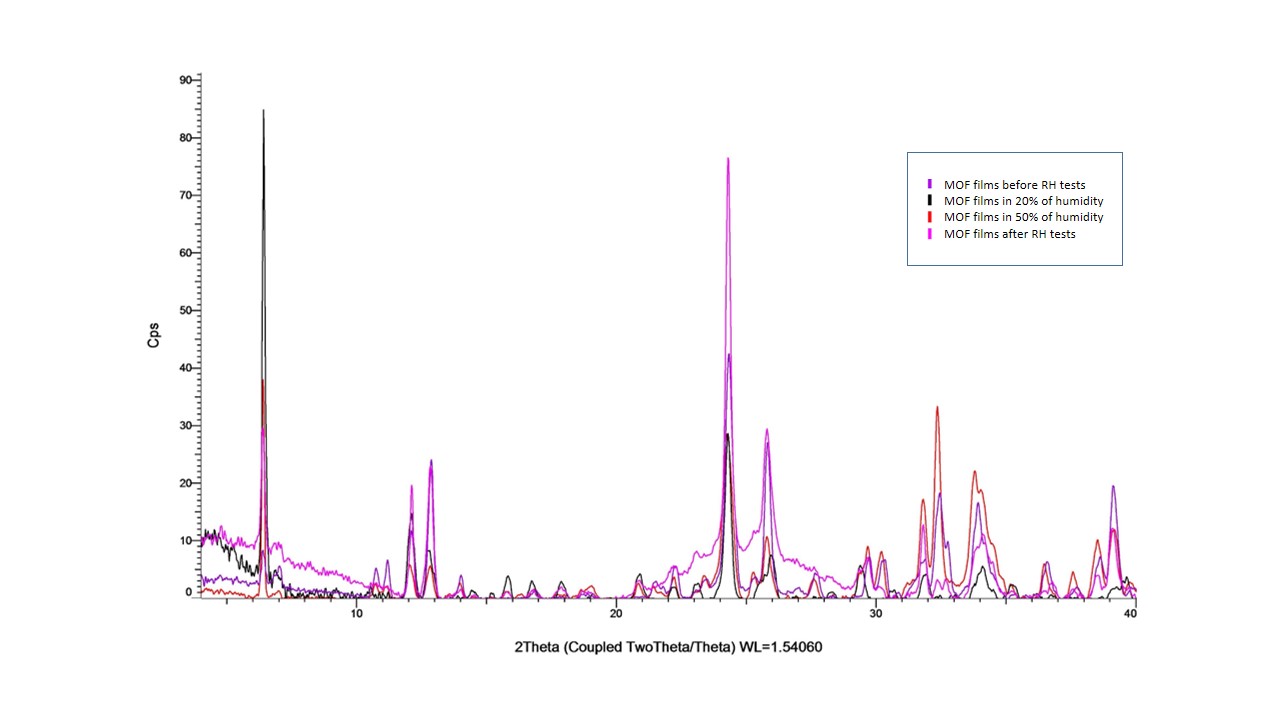


**Figure S1**. PXRD experiments on the MOF films before and after RH tests, and with 20 and 50% of humidity.


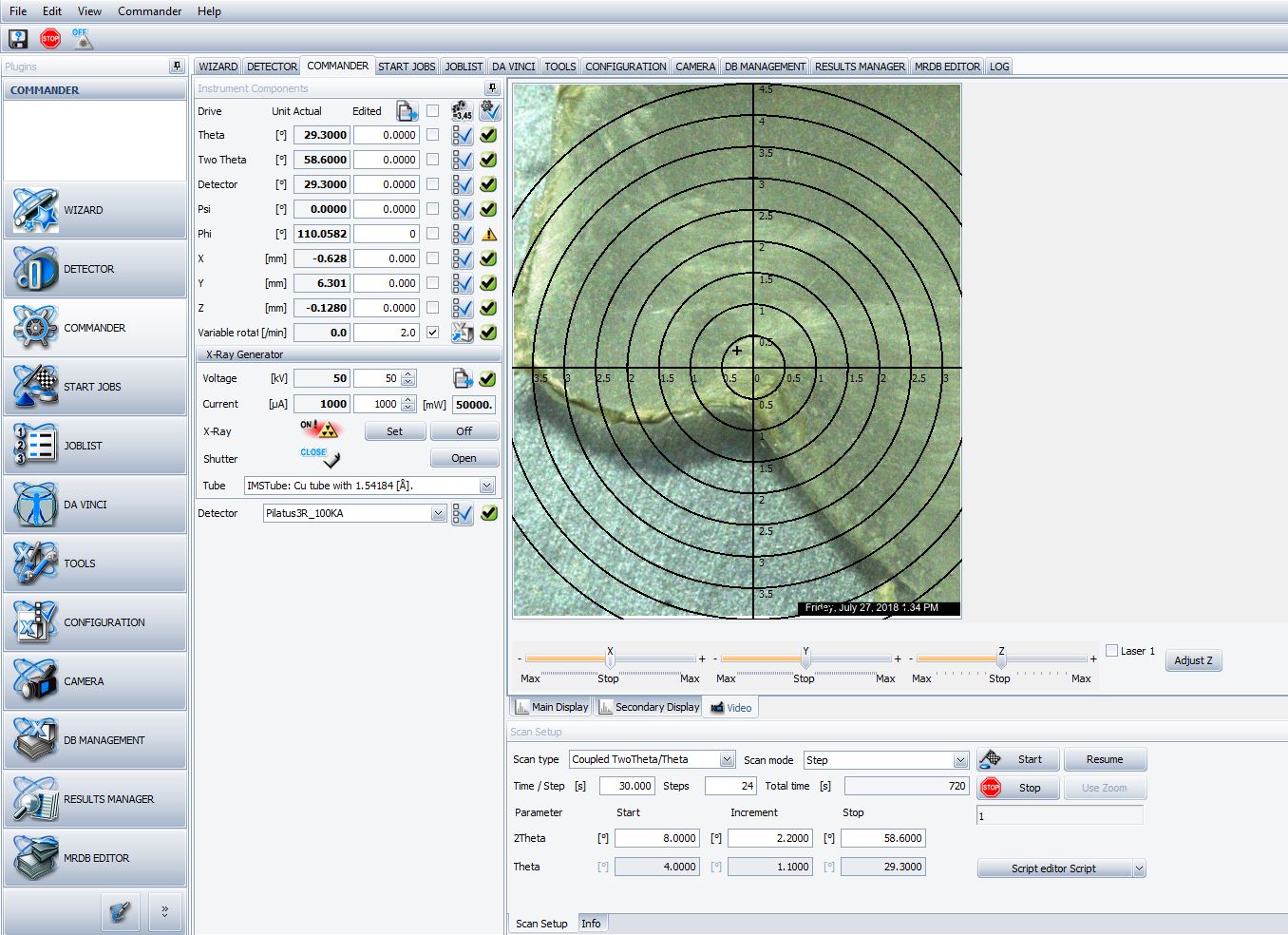


**Figure S2**. Screenshot of the analyzed part of the MOF film.

*Fluorescence measurements*

K-perylene displayed photoluminescent properties, arising from the perylene linker. The emission of the MOF is blue shifted (565 nm) in comparison to the protonated ligand (665 nm Figure S1). This is in agreement with the different stacking in both solids. The ordered structure in the MOF prevent the theperylene cores from aggregation, which leads to a red shift in the emission band as well as to the loss of its fine vibrational structure ^1^. With all, the fluorescence band is bathochromically shifted compared to the free tetracarboxylate unit in solution (485 nm Figure S2), which can be the effect of the coordination to the potassium cations.


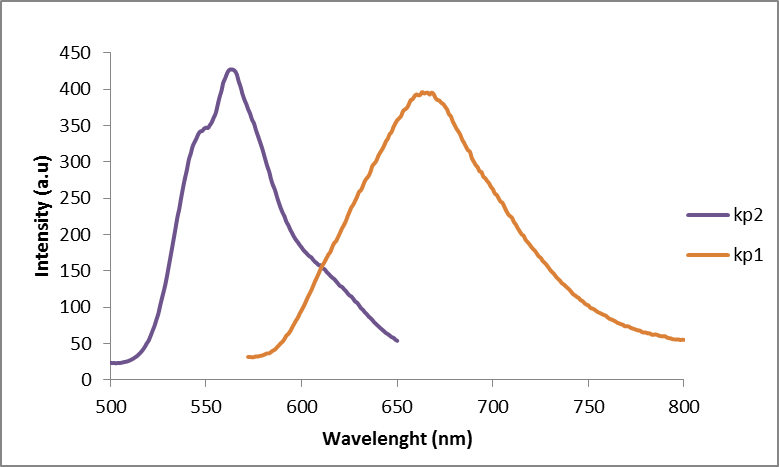


**Figure S3**. Emission spectra of Perylene-K (λ_exc_ = 410 nm) and 3,4,9,10-perylenetetracarboxylic acid (λ_exc_ = 585 nm) in the solid state.


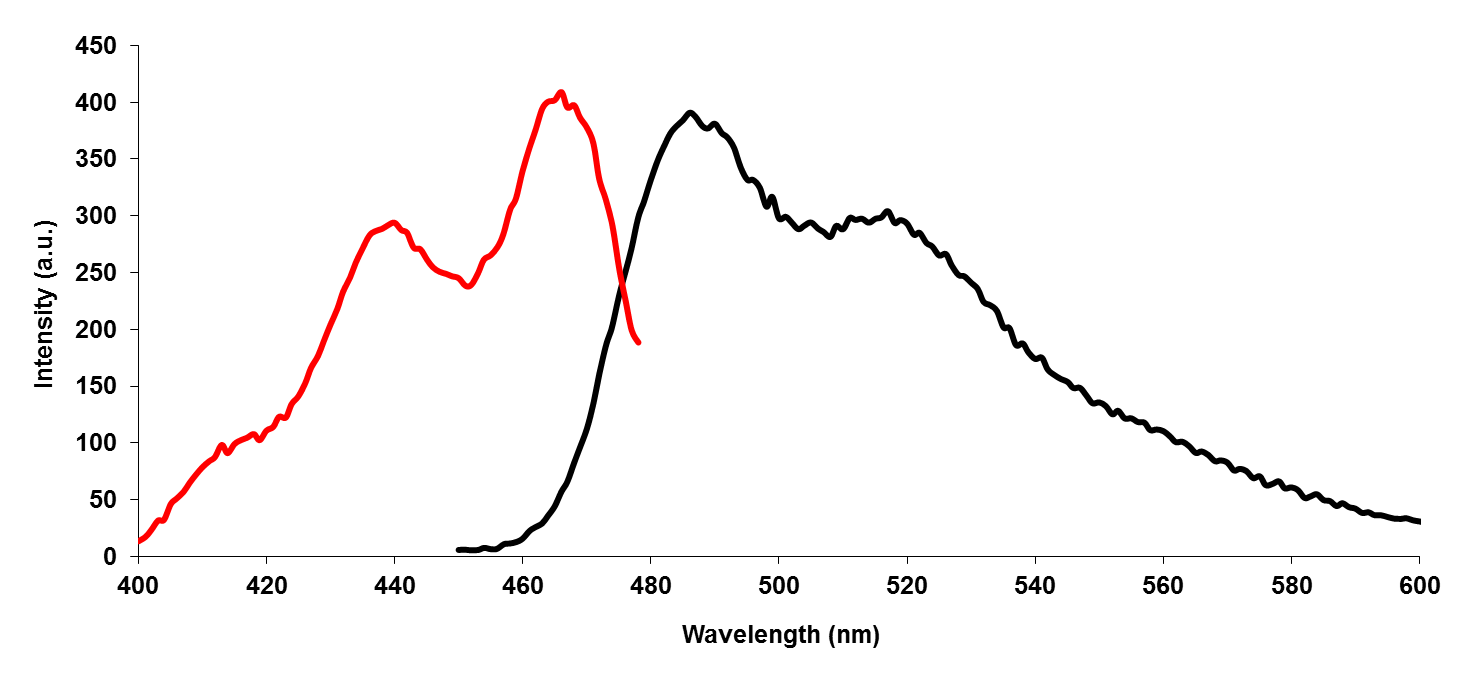


**Figure S4**. Absorption (red) and emission (black) bands of perylene-K after solution in water.

**
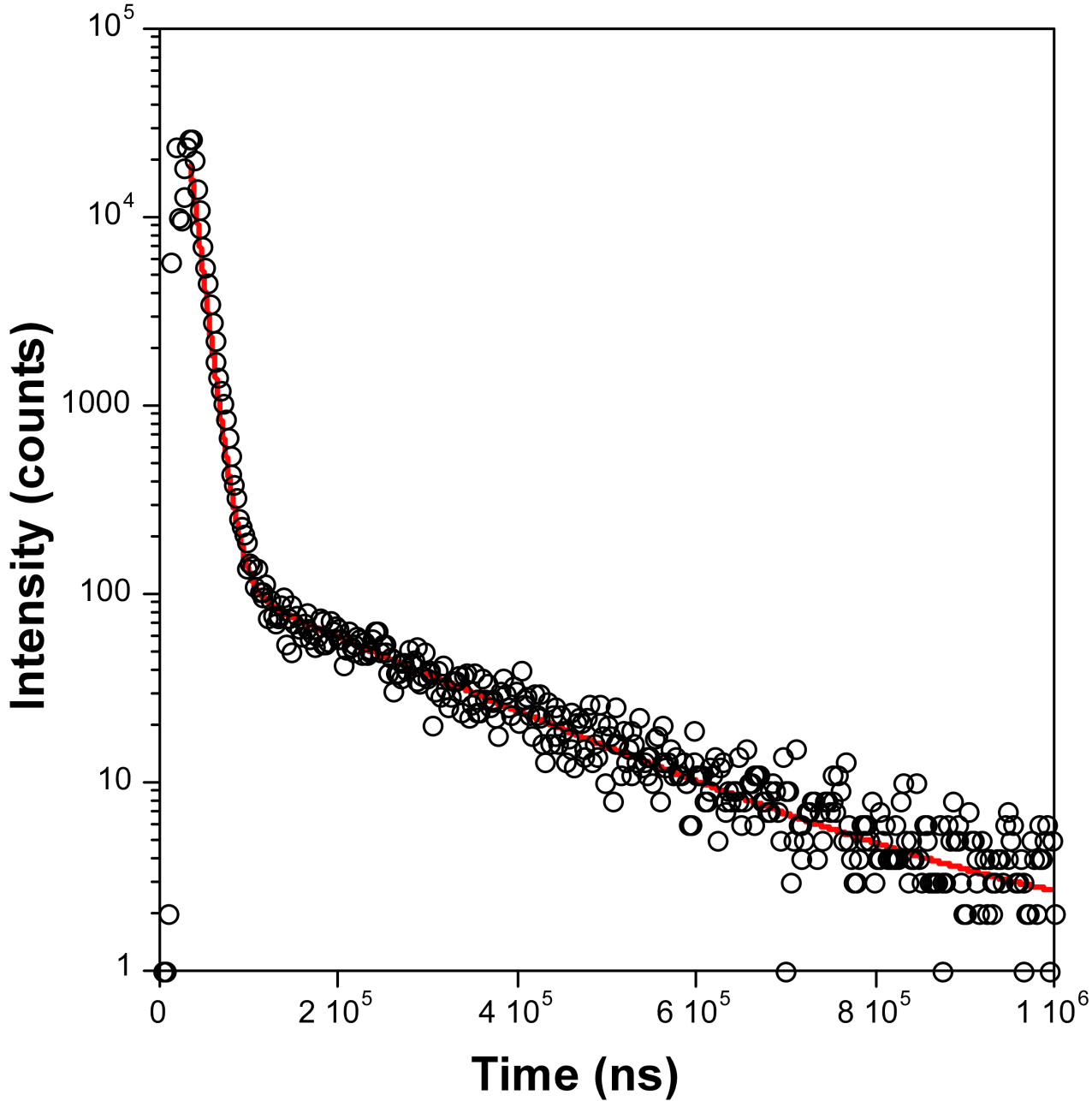
**

**Figure S5**. Tail fitting of the decay curve of compound **1**.

**SEM**

|   (a) |   (b) |
| --- | --- |
|   (c) | |

**Figure S6**. Scanning electron microscope image of the K-Pery on silver nanoparticle film at (a) 142X (100 µm scale); (b) 500X (20 µm scale bar) and (c) 1KX (10 µm scale bar) magnification.

**References**

1 Ito, F., Kogasaka, Y. & Yamamoto, K. Fluorescence spectral changes of perylene in polymer matrices during the solvent evaporation process. *The Journal of Physical Chemistry B* **117**, 3675-3681 (2013).
