# Supplementary material for: A Potassium Metal-Organic Framework based on Perylene-3,4,9,10-tetracarboxylate as Sensing Layer for Humidity Actuators
Source: Sci Rep. 2018 Sep 26;8:14414. doi: 10.1038/s41598-018-32810-7 (PMC6158245; doi:10.1038/s41598-018-32810-7)

# checkCIF/PLATON report

Structure factors have been supplied for datablock(s) Compound1

THIS REPORT IS FOR GUIDANCE ONLY. IF USED AS PART OF A REVIEW PROCEDURE FOR PUBLICATION, IT SHOULD NOT REPLACE THE EXPERTISE OF AN EXPERIENCED CRYSTALLOGRAPHIC REFEREE.

No syntax errors found.    CIF dictionary    Interpreting this report

## Datablock: Compound1

---

```
Bond precision:  C-C = 0.0048 A          Wavelength=0.71073

Cell:            a=12.1841(6)     b=12.1841(6)     c=14.0000(7)
                 alpha=90         beta=90          gamma=120
Temperature:     100 K
```

| | Calculated | Reported |
|---|---|---|
| Volume | 1799.9(2) | 1799.9(2) |
| Space group | P -3 | P -3 |
| Hall group | -P 3 | -P 3 |
| Moiety formula | 2(C36 H12 K4 O12), 7(O) | C72 H30 K8 O27, 4(H3O) |
| Sum formula | C72 H24 K8 O31 | C72 H46 K8 O31 |
| Mr | 1697.71 | 1715.88 |
| Dx,g cm-3 | 1.566 | 1.583 |
| Z | 1 | 1 |
| Mu (mm-1) | 0.570 | 0.570 |
| F000 | 856.0 | 874.0 |
| F000’ | 858.09 | |
| h,k,lmax | 16,16,18 | 16,16,18 |
| Nref | 3144 | 3142 |
| Tmin,Tmax | 0.897,0.966 | 0.589,0.846 |
| Tmin’ | 0.897 | |

```
Correction method= # Reported T Limits: Tmin=0.589 Tmax=0.846
AbsCorr = NUMERICAL

Data completeness= 0.999          Theta(max)= 28.761

R(reflections)= 0.0765( 2701)     wR2(reflections)= 0.1766( 3142)

S = 1.045                 Npar= 169
```

---

The following ALERTS were generated. Each ALERT has the format
**test-name_ALERT_alert-type_alert-level**.
Click on the hyperlinks for more details of the test.

---

## Alert level A

```
PLAT430_ALERT_2_A Short Inter D...A Contact  O1       ..O1W              2.50 Ang.
                                                  x,y,z  =      1_555 Check
```

**Author Response: This short distance correponds to a disordered hydrogen bond for which hydrogen atoms could not be refined.**

---

## Alert level B

```
PLAT043_ALERT_1_B Calculated and Reported Mol. Weight Differ by ..      18.17 Check
```

**Author Response: Hydrogen atoms belonging to both coordinated water and crystallisation hydronium molecules were not be placed because these molecules are disordered a hydrogen bonding scheme could not be unambigously determined. This brings a dis in the molecular weight.**

```
PLAT430_ALERT_2_B Short Inter D...A Contact  O1       ..O2W              2.67 Ang.
                                              -x+y,-x,z  =      3_555 Check
```

**Author Response: This short distance correponds to a disordered hydrogen bond for which hydrogen atoms could not be refined.**

```
PLAT430_ALERT_2_B Short Inter D...A Contact  O3       ..O2W              2.61 Ang.
                                                  x,y,z  =      1_555 Check
```

**Author Response: This short distance correponds to a disordered hydrogen bond for which hydrogen atoms could not be refined.**

---

## Alert level C

```
CHEMW01_ALERT_1_C  The difference between the given and expected weight for
            compound is greater 1 mass unit. Check that all hydrogen
            atoms have been taken into account.
DIFMN02_ALERT_2_C  The minimum difference density is < -0.1*ZMAX*0.75
            _refine_diff_density_min given =     -1.855
            Test value =     -1.425
DIFMN03_ALERT_1_C  The minimum difference density is < -0.1*ZMAX*0.75
            The relevant atom site should be identified.
DIFMX02_ALERT_1_C  The maximum difference density is > 0.1*ZMAX*0.75
            The relevant atom site should be identified.
PLAT041_ALERT_1_C Calc. and Reported SumFormula    Strings  Differ     Please Check
PLAT068_ALERT_1_C Reported F000 Differs from Calcd (or Missing)...     Please Check
PLAT097_ALERT_2_C Large Reported Max.  (Positive) Residual Density       1.90 eA-3
PLAT098_ALERT_2_C Large Reported Min.  (Negative) Residual Density      -1.86 eA-3
PLAT213_ALERT_2_C Atom K1              has ADP max/min Ratio .....        3.2 prolat
PLAT213_ALERT_2_C Atom O2              has ADP max/min Ratio .....        3.6 prolat
PLAT250_ALERT_2_C Large U3/U1 Ratio for Average U(i,j) Tensor ....        3.2 Note
```

```
PLAT340_ALERT_3_C Low Bond Precision on  C-C Bonds ...............    0.00479 Ang.
PLAT906_ALERT_3_C Large K Value in the Analysis of Variance ......      7.951 Check
PLAT971_ALERT_2_C Check Calcd Resid. Dens.  0.84A   From K1             1.84 eA-3
PLAT972_ALERT_2_C Check Calcd Resid. Dens.  0.84A   From K1            -1.75 eA-3
PLAT975_ALERT_2_C Check Calcd Resid. Dens.  0.87A   From O3W            0.51 eA-3
```

---

## Alert level G

```
FORMU01_ALERT_1_G  There is a discrepancy between the atom counts in the
            _chemical_formula_sum and _chemical_formula_moiety. This is
            usually due to the moiety formula being in the wrong format.
            Atom count from _chemical_formula_sum:   C72 H46 K8 O31
            Atom count from _chemical_formula_moiety:C72 H42 K8 O31
FORMU01_ALERT_2_G  There is a discrepancy between the atom counts in the
            _chemical_formula_sum and the formula from the _atom_site* data.
            Atom count from _chemical_formula_sum:C72 H46 K8 O31
            Atom count from the _atom_site data:  C72 H24 K8 O31.00460
CELLZ01_ALERT_1_G Difference between formula and atom_site contents detected.
CELLZ01_ALERT_1_G WARNING: H atoms missing from atom site list. Is this intentional?
           From the CIF: _cell_formula_units_Z    1
           From the CIF: _chemical_formula_sum  C72 H46 K8 O31
           TEST: Compare cell contents of formula and atom_site data

           atom    Z*formula  cif sites diff
           C         72.00     72.00    0.00
           H         46.00     24.00   22.00
           K          8.00      8.00    0.00
           O         31.00     31.00    0.00
PLAT004_ALERT_5_G Polymeric Structure Found with Maximum Dimension          3 Info
PLAT042_ALERT_1_G Calc. and Reported MoietyFormula Strings  Differ     Please Check
PLAT083_ALERT_2_G SHELXL Second Parameter in WGHT  Unusually Large       7.41 Why ?
PLAT300_ALERT_4_G Atom Site Occupancy of O1W        Constrained at        0.5 Check
PLAT300_ALERT_4_G Atom Site Occupancy of O2W        Constrained at     0.3333 Check
PLAT300_ALERT_4_G Atom Site Occupancy of O3W        Constrained at     0.3333 Check
PLAT302_ALERT_4_G Anion/Solvent/Minor-Residue Disorder (Resd  2  )       100% Note
PLAT302_ALERT_4_G Anion/Solvent/Minor-Residue Disorder (Resd  3  )       100% Note
PLAT302_ALERT_4_G Anion/Solvent/Minor-Residue Disorder (Resd  4  )       100% Note
PLAT311_ALERT_2_G Isolated Disordered Oxygen Atom (No H's ?) .....        O1W Check
PLAT311_ALERT_2_G Isolated Disordered Oxygen Atom (No H's ?) .....        O2W Check
PLAT311_ALERT_2_G Isolated Disordered Oxygen Atom (No H's ?) .....        O3W Check
PLAT333_ALERT_2_G Large Aver C6-Ring C-C Dist. C8       -C10             1.45 Ang.
PLAT764_ALERT_4_G Overcomplete CIF Bond List Detected (Rep/Expd) .       1.83 Ratio
PLAT774_ALERT_1_G Suspect X-Y Bond in CIF: K2       --K1        ..       4.39 Ang.
PLAT774_ALERT_1_G Suspect X-Y Bond in CIF: K2       --K1        ..       4.39 Ang.
PLAT774_ALERT_1_G Suspect X-Y Bond in CIF: K2       --K1        ..       4.39 Ang.
PLAT779_ALERT_4_G Suspect or Irrelevant (Bond) Angle in CIF .... #        147 Check
              O3   -C2   -K1      1.555   1.555   2.000            41.18 Deg.
PLAT789_ALERT_4_G Atoms with Negative _atom_site_disorder_group  #          3 Check
PLAT790_ALERT_4_G Centre of Gravity not Within Unit Cell: Resd.  #          4 Note
              O
PLAT802_ALERT_4_G CIF Input Record(s) with more than 80 Characters          2 Info
PLAT910_ALERT_3_G Missing # of FCF Reflection(s) Below Theta(Min).          2 Note
PLAT961_ALERT_5_G Dataset Contains no Negative Intensities .......     Please Check
PLAT978_ALERT_2_G Number C-C Bonds with Positive Residual Density.          7 Info
```

---

1 **ALERT level A** = Most likely a serious problem - resolve or explain
3 **ALERT level B** = A potentially serious problem, consider carefully
16 **ALERT level C** = Check. Ensure it is not caused by an omission or oversight
28 **ALERT level G** = General information/check it is not something unexpected

13 ALERT type 1 CIF construction/syntax error, inconsistent or missing data

```
19 ALERT type 2 Indicator that the structure model may be wrong or deficient
 3 ALERT type 3 Indicator that the structure quality may be low
11 ALERT type 4 Improvement, methodology, query or suggestion
 2 ALERT type 5 Informative message, check
```

---

It is advisable to attempt to resolve as many as possible of the alerts in all categories. Often the minor alerts point to easily fixed oversights, errors and omissions in your CIF or refinement strategy, so attention to these fine details can be worthwhile. In order to resolve some of the more serious problems it may be necessary to carry out additional measurements or structure refinements. However, the purpose of your study may justify the reported deviations and the more serious of these should normally be commented upon in the discussion or experimental section of a paper or in the "special_details" fields of the CIF. checkCIF was carefully designed to identify outliers and unusual parameters, but every test has its limitations and alerts that are not important in a particular case may appear. Conversely, the absence of alerts does not guarantee there are no aspects of the results needing attention. It is up to the individual to critically assess their own results and, if necessary, seek expert advice.

**Publication of your CIF in IUCr journals**

A basic structural check has been run on your CIF. These basic checks will be run on all CIFs submitted for publication in IUCr journals (*Acta Crystallographica*, *Journal of Applied Crystallography*, *Journal of Synchrotron Radiation*); however, if you intend to submit to *Acta Crystallographica Section C* or *E* or *IUCrData*, you should make sure that full publication checks are run on the final version of your CIF prior to submission.

**Publication of your CIF in other journals**

Please refer to the *Notes for Authors* of the relevant journal for any special instructions relating to CIF submission.

---

**PLATON version of 14/07/2018; check.def file version of 05/06/2018**

**Datablock Compound1** - ellipsoid plot

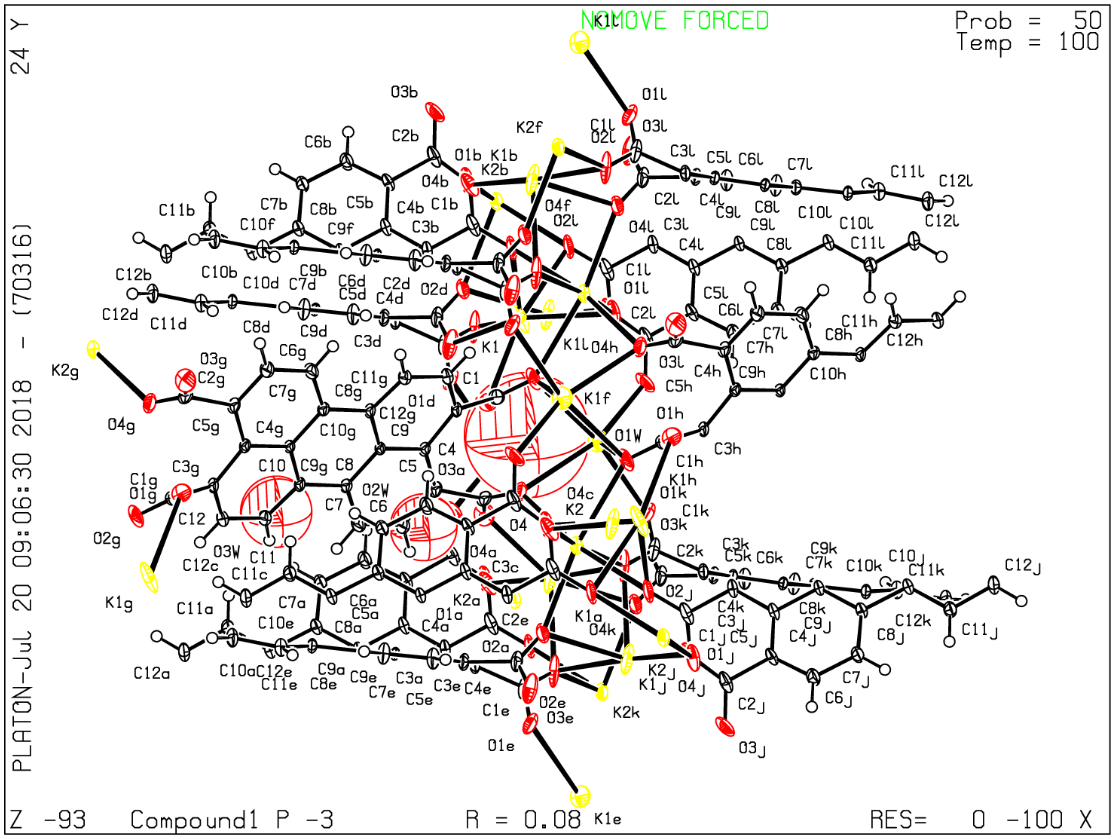

Supplement: Supplementary file 2 — Compound 1 [file 41598_2018_32810_MOESM2_ESM.pdf]
